# Ethylene in fruits: beyond ripening control

**DOI:** 10.1093/hr/uhae229

**Published:** 2024-08-09

**Authors:** Wei Huang, Cong Tan, Hongwei Guo

**Affiliations:** State Key Laboratory of Agricultural Genomics, Key Laboratory of Genomics, Ministry of Agricultural, BGI Research, Shenzhen 518083, China; BGI Bioverse, Shenzhen 518083, China; BGI Bioverse, Shenzhen 518083, China; New Cornerstone Science Laboratory, Institute of Plant and Food Science, Department of Biology, School of Life Sciences, Southern University of Science and Technology (SUSTech), Shenzhen, Guangdong 518055, China; Shenzhen Branch, Guangdong Laboratory for Lingnan Modern Agriculture, Agricultural Genomics Institute at Shenzhen, Chinese Academy of Agricultural Sciences, Shenzhen 518120, China

## Abstract

Fruits are a rich source of nutrients, minerals, and dietary fibers for both humans and animals. While the gaseous phytohormone ethylene is well-known for its role in controlling fruit ripening, there is growing evidence that ethylene also plays crucial roles in regulating other developmental processes of fruits, such as sex determination, fruit set, and fruit growth. In this review, we aim to revisit these findings from various species like cucumber, melon, tomato, rice, maize, and more. These studies not only enhance our understanding of ethylene’s function in fruits but also highlight the potential for manipulating ethylene to improve crops. Furthermore, we discuss recent studies that show the ethylene precursor ACC (1-AMINOCYCLOPROPANE-1-CARBOXYLATE), and the ethylene signaling components EIN2 (ETHYLENE INSENSITIVE2) and EIN3 (ETHYLENE INSENSITIVE3) have ethylene-independent function in specific conditions. This phenomenon, combined with findings of dosage-dependent ethylene functions in certain conditions, highlights the importance of analyzing mutants with completely blocked ethylene pathways in different species at specific developmental stages and tissue types. Overall, this review offers a timely and essential summary of ethylene’s role in sex determination, fruit formation, and fruit growth, which could be beneficial for horticulture crop breeding.

## Introduction

Flowering plants have evolved an elegant strategy for fruit development: during the growth stage, the unripe fruits are usually hard and unattractive to predators, providing seed protection. After seed development and fruit growth have been completed, a highly coordinated regulatory network involving phytohormones triggers alterations in fruit appearance, texture, flavor, and aroma, making fruits become attractive and edible to frugivores so that seed dispersal occurs as an accompaniment. Generally, the making of a fruit comprises three stages: fruit set, growth, and ripening. In the case of dioecious species, there is an additional but critical stage before fruit set: sex determination.

Literature has demonstrated that phytohormones plays a crucial role in the molecular regulation of all stages of fruit development. After fruit set, both dry fruits like *Arabidopsis* and fleshy fruits like tomato undergo cell division and expansion, which are essential for determining fruit size before maturation. Auxin and gibberellin (GA) have been identified as key regulators of cell division and expansion, respectively [1]. To initiate fruit set at the appropriate time, the preanthesis ovaries remain in a phase of dormancy, during which cell division activities are inhibited by the negative regulators of auxin signaling such as IAA9, ARF7, and the negative regulator of GA signaling DELLA [2–4]. Successful fertilization triggers an increase in auxin and GA content, leading to activated cell division and expansion in young fruits. Recently, the involvement of SlDELLA and the SlARF7/SlIAA9 complex in mediating crosstalk between auxin and GA pathways during fruit set and growth has been well characterized in tomato [5, 6]. There is a significant interaction between ethylene and auxin in various stages of the fruit set process [7]. Furthermore, the control of fruit maturation/ripening is influenced by various phytohormones in different species: auxin seems to be the primary regulator of fruit maturation in Arabidopsis, as a regulated auxin minimum is necessary for seed dispersal of Arabidopsis siliques [8]; fruit ripening in non-climacteric fruits like strawberry and in climacteric fruits like tomato appears to be primarily achieved by abscisic acid (ABA) and ethylene, respectively [9–11], although there are numerous interactions between these two hormones in both climacteric and non-climacteric fruits [12–14]. Through a genome-wide association study (GWAS) of fruit firmness in the 266 tomato core accessions, *SlEIN4*, an ethylene receptor gene, was identified as the key factor governing fruit firmness in tomato [15]. Additionally, other phytohormones including auxin, jasmonic acid (JA), gibberellin, and brassinosteroid (BR) have been implicated in the fine-tuning fruit ripening control by modulating ethylene biosynthesis in most cases. The ethylene response factor ERF.D7 activates the auxin response factor ARF2 to regulate fruit ripening in tomato [16, 17]. During fruit ripening, the accumulation of carotenoids is modulated by the auxin-ethylene balance [18]. The jasmonate-activated transcription factor MdMYC2 promotes fruit ripening by activating ethylene biosynthesis in apple [19]. In tomato, gibberellin participates in fruit ripening and softening by mediating multiple hormonal signals [20]. The BR biosynthetic gene SlCYP90B3 positively regulates fruit ripening via an ethylene-dependent pathway [21].

Owing to its fundamental role in ripening control of climacteric fruits, ethylene is the most widely explored phytohormone in fruit biology. Ancient Chinese people burned incense to ripen pears, and early Egyptians gashed figs to induce their ripening. These are the two earliest records of manipulating ethylene, despite the underlying mechanism not being elucidated until thousands of years later [22]. Over the past decades, the characterization of mutants and transgenic plants corresponding to ethylene biosynthesis, perception, and response pathways in *Arabidopsis* and tomato has greatly enhanced our understanding of the molecular basis of ethylene in fruit quality and ripening control [23]. Based on the autoinhibitory and autocatalytic features, McMurchie *et al*. [24] introduced two unique ethylene systems: System-1 represents the basal low level of ethylene production in preclimacteric fruits, vegetative tissues, and nonclimacteric fruits. System-2, on the other hand, is the high-level ethylene production observed during ripening in climacteric fruits and in certain senescent flowers [25]. Despite the extensively studied function of system-2 ethylene in fruit ripening control, emerging evidence shows that system-1 ethylene is essential for fruit set and growth, as well as sex determination in dioecious plants.

## Ethylene in sex determination

Approximately 5–6% of flowering plant species produce unisexual flowers, representing a model system to unveil mechanisms of sex determination in plants. The successive cloning of genes underlying the genetic ‘FAM’ model in cucumber (*Cucumis sativus*) has made the ‘one-hormone hypothesis’ convincing and widely accepted [26, 27]. The hypothesis posits ethylene as the most important regulator of unisexual flower development in cucumber.

With map-based cloning, it was found that all the ‘sex genes’ of cucumber, including *F* (*Female*, *CsACS1G*), *M* (*Monoecious*, *CsACS2*), and *A* (*Androecious*, *CsACS11*), encode 1-aminocyclopropane-1-carboxylate synthase (ACS), which catalyzes the rate-limiting step in ethylene biosynthesis. The nature of gynoecious cucumbers conferred by the *F* locus has been the most used accession for breeding high-yield varieties for half a century. The gain-of-function (GOF) of a duplicated copy of *CsACS1G*, but not *CsACS1* per se or other genes, in the *F* locus is responsible for the development of all-female flowers [28, 29]. *ACS1G* in gynoecious cucumbers shows a distinct structural variation of the genome, demonstrating a different promoter and expression level than that of *ACS1* in monoecious cucumbers. The higher *CsACS1G* transcripts increase ethylene content, leading to all-female flowers [30]. Regardless of the breeding value of *CsACS1G* in accessions containing the *F* locus, *CsACS1* plays a less important role than *M*/*CsACS2* and *A*/*CsACS11* in sex determination. *CsACS11* acts together with *CsACO2* at an early stage of floral development, regulating carpel development of female flowers. Dysfunction of either *CsACS11* or *CsACO2* leads to the loss of female flowers [31, 32]. This is also the case for *CmACS11* and *CmACO3* in melon (*Cucumis melon*) and *CpACO*1A in zucchini (*Cucurbita pepo*) [33, 34]. Interestingly, *M*/*CsACS2* mediates a positive feedback regulation of secondary ethylene biosynthesis. It is believed that the amplification of enough ethylene is crucial for the arrest of stamen primordia development, as *CsACS2* knock-down plants display bisexual flowers [33, 34]. Recent studies have revealed that two ethylene responsive factors (ERF) in cucumber, CsERF110 and CsERF31, are involved in the positive feedback loop through directly activating the transcription of *CsACS11* and *CsACS2*, respectively [35, 36]. Kock-down of *CsERF3*1 in cucumber gynoecy line causes defective bisexual flowers, replacing the female flowers [36]. It is noteworthy that the *A* (*Andromonoecy*) locus in melon is encoded by an *ACS* family member designated as *CmACS7*, which inhibits the development of the male organs and is not required for carpel development [33]. The melon *A*/*CmACS7* is a close homolog of the cucumber *M*/*CsACS2* gene, and these two genes share a conserved function in regulating the andromonoecious phenotype of cucumber and melon [37]. Furthermore, two independent studies found that the watermelon (*Citrullus lanatus*) *A* locus gene *A*/*CitACS4* is required for monoecy/andromonoecy regulation. Reduced ethylene production in the floral buds by a missense mutation of *CitACS4* leads to the conversion of female into hermaphrodite flowers, and therefore of monoecy into andromonoecy [38, 39].

Notably, *CsWIP1*, which encodes a C2H2 zinc-finger-type transcription factor, plays an essential role in sex determination. Its ortholog *CmWIP1* in melon indirectly represses the expression of the andromonoecious gene *A*/*CmACS7* [40]. Gynoecious plants can be obtained via gene-editing of *CsWIP1* and *ClWIP1* in monoecious plants of cucumber and watermelon, respectively [41, 42]. In cucumber, *CsWIP1* is suppressed by *CsACS11* and controls the coexistence of male and female flowers in monoecious plants by acting as a carpel inhibitor [31]. CsWIP1 inhibits *CsACO2* expression by binding to its promoter, leading to pistil abortion [31]. CsWIP1 further suppresses *CsACS2* expression, resulting in the derepression of stamen development inhibition [31]. Interestingly, in melon, it was found that the female-promoting gene, *CmACS11*, represses the expression of the male-promoting gene *CmWIP1* via deposition of H3K27me3 [43]. Further study revealed that CmWIP1 promotes male flower development by recruiting a corepressor TOPLESS to inhibit the carpel identity gene *CRC* (*CRABS CLAW*) expression through histone deacetylation [44].

**Figure 1:**
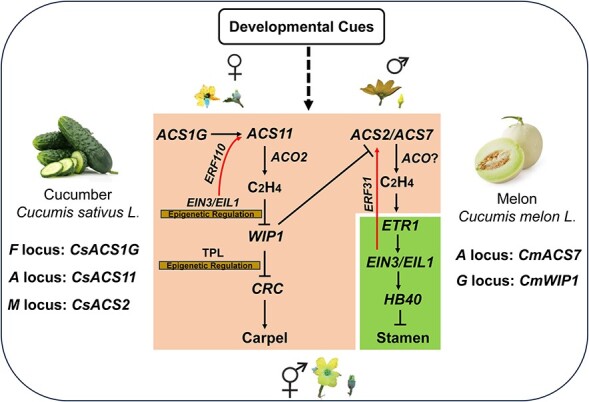
A working model demonstrating the involvement of ethylene pathway in sex determination in Cucurbitaceae species. At different flowering stages, *ACS1G,* which only presented in cucumber, and *ACS11* synergistically with *ACO2* to produce endogenous ethylene, which suppresses *WIP1* expression. WIP1 can inhibit carpel development by repressing the *ACS2/ACS7* expression, which in turn inhibits stamen development. To amplify enough ethylene for the arrest of stamen primordia development, ERF31 and ERF110 mediate the positive feedback regulation via directly activating the transcription of *ACS2/ACS7* and *ACS11*, respectively. The transcription factor genes *CRC* and *HB40* act as carpel initiator and stamen inhibitor at the downstream of WIP1 and EIN3/EIL1, respectively. The accumulation of *WIP1* and *CRC* transcripts are associated with the ethylene-mediated histone methylation and deacetylation, respectively. To date, there are three locus (*F/CsACS1G*, *M/CsACS12*, *A/CsACS2*) and two locus (*A/CmACS7*, *G/CmWIP1*) controlling sex determination that have been map-cloned in cucumber and melon, respectively. The regular arrow indicates positive regulation, and the ‘T’ represents negative regulation. The red arrow indicates positive feedback regulation.

Compared to ethylene biosynthesis genes, few mutants related to ethylene signaling components have been identified in dioecious species. Two semi-dominant ethylene-insensitive mutants in *C. pepo*, *Cpetr1a* and *Cpetr2b*, were recently discovered through ethyl methanesulfonate (EMS)-generated mutant screening. A comprehensive characterization of single and double mutants revealed that *CpETR* genes collaborate in controlling female flower determination. The proportion of male flowers is positively correlated with the degree of ethylene insensitivity, which depends on the dosage of *CpETR*-gene mutant alleles [45, 46]. It has been suggested that different levels of ethylene responses in stamen or carpel primordia are associated with the selective arrest during sex determination [47]. Ethylene produced in melon carpel is perceived in the stamen primordia through spatially differentially expressed ethylene receptors. Subsequently, the *CmEIN3/CmEIL1* genes in stamen primordia activate the expression of *CmHB40* to inhibit stamen development [48]. In contrast, ethylene in cucumber could induce female flower development through an anther-specific DNA damage process caused by the stamen preferential downregulation of the ethylene receptor gene *CsETR1* [49]. *CsAP3*, encoding a MADS-box transcription factor, is responsible for the organ preferential downregulation of *CsETR1* [50]. Finally, a calcium-dependent DNase gene, *CsCaN*, is activated by ethylene and is associated with the anther-specific DNA damage [51]. These findings reveal that ethylene is essential for sex determination in Cucurbitaceae species, involving ethylene biosynthesis and signal transduction genes at both the transcriptional regulation and the epigenetic regulation levels (Fig. 1).

It is noteworthy that some other hormones also play a role in sex determination in Cucurbitaceae species. The treatment of BR to cucumber results in both earlier and increased levels of female flower production. This effect also appears to depend on ethylene production and sensitivity, suggesting that BR acts upstream of ethylene during floral sex determination [52]. In contrast, GA induces the formation of male flowers in gynoecious cucumber, possibly by down-regulating ethylene biosynthesis and signaling genes in the apical shoot [53]. Intriguingly, the predominant role of ethylene in sex determination seems to be unique to Cucurbitaceae species. For instance, in maize, JA plays a predominant role in sex determination. Several maize *tasselseed* (*ts*) mutants affecting JA biosynthesis or catabolism, including *ts1*, *ts2*, *ts5*, exhibit a reversal in sex determination, resulting in generation of seeds in tassels [54]. Additionally, maize BR biosynthesis mutants demonstrate feminized tassels [55, 56]. These findings indicate that the function of ethylene in plant reproductive stages has extensive crosstalk with other phytohormones, and elucidating the interplay conferred by different hormones in sex determination merits further investigation.

## Ethylene in fruit set

The involvement of ethylene in pollination and fertilization processes has been a long-term topic in plant reproductive biology. There is a transient ethylene burst (<12 h) in tomato ovaries upon pollination, which is proposed to facilitate senescence of certain floral organs [57, 58]. The pollination process stimulates the expression of the ethylene biosynthetic genes in a developmentally regulated and tissue-specific manner, which is important for ovary/ovule and gametophyte development in orchid flowers [59, 60]. In *Nicotiana attenuata*, a self-compatible wild tobacco accession, the post-pollination ethylene burst is recognized as the harbinger of non-random mate selection [61]. In fact, ethylene acts as a suppressor of the self-incompatibility response. Treating stigmas with ethylene or suppressing the expression of a negative regulator of ethylene signaling, *CTR1* (*CONSTITUTIVE TRIPLE RESPONSE 1*), broke down the self-incompatibility in *Brassica rapa* [62]. During pollination, ethylene signaling modulates pollen tube growth through modifications of cell wall remodeling and calcium gradient in tomato [63]. Tomato pollen tubes in *etr3*-KO (knock out), a loss-of-function (LOF) mutant, and *Nr* (*Never ripe*), a GOF mutant, grow faster and slower than in WT, respectively [63]. In *Arabidopsis*, it was shown that ethylene promotes pollen tube growth by affecting actin filament organization via the cGMP-dependent pathway [64]. Upon successful fertilization, ethylene emissions and transcripts of ethylene biosynthesis/signaling genes decrease in tomato ovaries/young fruits [65, 66], suggesting a negative role of ethylene in fruit set. In accordance with that, the application of 1-MCP, an ethylene perception inhibitor, results in parthenocarpic tomato fruits [67]. As a typical self-pollination flowering plant, tomato stigmas wrapped by stamens at the anthesis stage ensure precise pollen spread. *Sletr1–1*, a GOF mutant of the tomato ethylene receptor gene, exhibits protruded stigmas and parthenocarpic fruits [67]. It was further demonstrated that ethylene prevents excess growth of tomato ovaries/stigmas at the anthesis stage by modulating GA biosynthesis/metabolism [67]. Consistently, the LOF mutant *slein2* also yields a large proportion of facultative parthenocarpic fruits in tomatoes [68]. Together, this evidence shows that upon pollination, ethylene might confer distinct functions before and after fertilization. Because the time of pollination and the subsequent fertilization are linear, it is suggested that we should make a distinction when dissecting the role of ethylene at different periods during fruit set.

**Figure 2:**
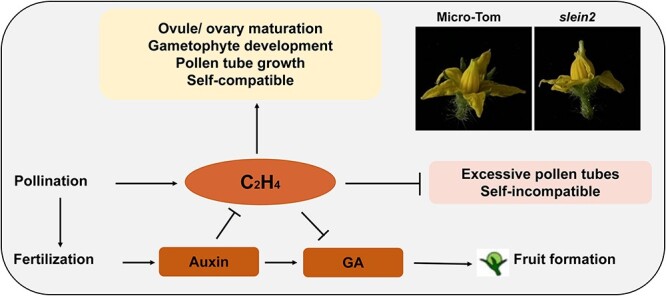
Model of the ethylene-mediated fruit set. In flowering plant, ethylene promotes selfing (self-compatible) while inhibiting outcrossing (self-incompatible). The pollination process stimulates a transient ethylene burst, which plays a positive role in the maturation of the reproductive organs and pollen tube growth for the following fertilization. Successful fertilization induces the auxin pathway that can downregulate ethylene biosynthesis, leading to derepressed GA pathway and thereby initiates fruit formation. The upper right corner photos show the extruded stigma in tomato *slein2* LOF mutant. The regular arrow indicates positive regulation, and the ‘T’ represents negative regulation.

To ensure successful fertilization, the ethylene signal is crucial for both attracting the pollen tube and preventing polytubey in the female gametophyte/ovule. The over-accumulation of EIN3 in *ebf1 ebf2* synergid cells leads to the failure of pollen tube guidance, possibly by activating a sugar transporter gene, *SENESCENCE-ASSOCIATED GENE29* (*SAG29*/*SWEET15*) [69]. When the pollen tube reaches the ovule, it delivers sperm cells to execute the double fertilization of two female gametophytes: the egg cell and the central cell. Fertilization of the central cell induces the synergid-endosperm fusion, which causes rapid dilution of pre-secreted pollen tube attractant in the persistent synergid cell. This process also leads to selective disorganization of the synergid nucleus during endosperm proliferation, preventing the attractions of an excess number of pollen tubes [70]. Meanwhile, fertilization of the egg cell predominantly activates ethylene signaling, which induces the disorganization of the synergid nucleus [70]. *Arabidopsis ein2* and *ein3 eil1* mutants show persistent synergid presence and attraction of extra pollen tubes [71], suggesting that ethylene signaling is essential for linking fertilization to synergid breakdown and the formation of a pollen tube barrier. Analysing these three findings suggests that there is a subtle dosage effect of EIN3 protein in controlling plant fertility, as mutants with either enhanced accumulation of EIN3 or deletion of EIN2-EIN3/EIL1 show compromised fertilization in *Arabidopsis* [69–71].

Very recently, however, by generating a CRISPR/Cas9 LOF quintuple mutation of all five 1-AMINOCYCLOPROPANE-1-CARBOXYLATE OXIDASE (ACO) coding genes, which are responsible for converting ACC to ethylene gas *in vivo*, Li *et al.* [72] found that the lack of ethylene does not affect reproductive success and synergid cell death in *Arabidopsis*. The finding, together with observations in *ein2* and *ein3eil1* mutants, suggests that certain components of ethylene signaling, including EIN2 and EIN3/EIL1, but not ethylene per se, are involved in the degeneration of the persistent synergid cell [70–72]. In fact, both EIN2 and EIN3 have recently been proposed to mediate ethylene-independent signaling pathways. EIN2 was reported to participate in glucose-mTOR signaling to mediate growth in *Arabidopsis* [73]. EIN3 confers an ethylene-independent function of synergid cell death upon fertilization in *Arabidopsis* [74]. Moreover, the ethylene precursor ACC also exhibits an ethylene-independent function in controlling pollen tube attraction, as *ACS* octuplet mutant plants demonstrate defects in pollen tube attraction and fertility, which could not be rescued by ethylene gas treatment [75].

**Figure 3:**
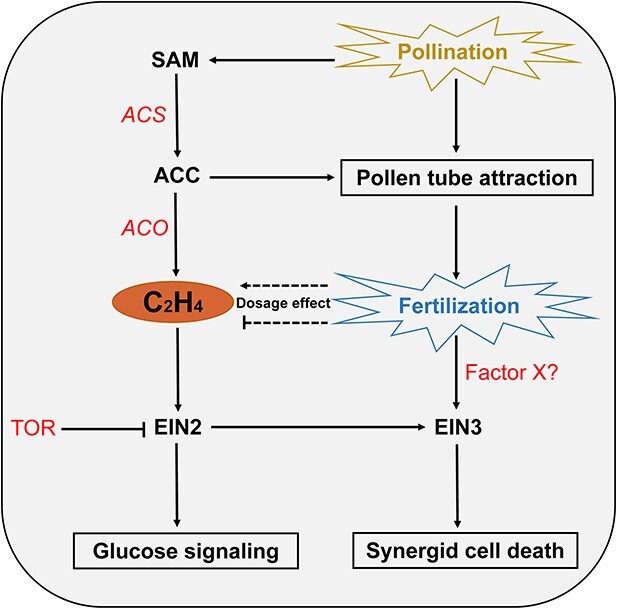
Schematic diagram of ethylene-independent function of ACC, EIN2, and EIN3 in Arabidopsis. Upon pollination, ACC functions in pollen tube attraction in an ethylene-independent manner, as the failure of pollen tube attraction in *ACS* octuplet mutant could not be rescued by ethylene gas treatment [75]. The successful fertilization depends on the dosage effect of the ethylene signaling, as either enhanced accumulation of EIN3 or deletion of EIN2-EIN3/EIL1 shows compromised fertilization [70–72]. In parallel, fertilization stimulates EIN3 accumulation to trigger synergid cell death by an unknown factor [74]. Besides, EIN2, the central component of the ethylene pathway, participates in the mTOR-glucose signaling pathway in an ethylene-independent manner [73], while it needs further investigation to see whether the mTOR-EIN2-glucose signaling module gets involved in fruit set. The regular arrow indicates positive regulation, and the ‘T’ represents negative regulation.

For a better understanding of the ethylene function during fruit set, we have integrated all these findings in Figs 2 and 3 in the present review. Given that all these ethylene-independent functions are deciphered in the model plant *Arabidopsis*, our knowledge of the role of ethylene pathway genes in fleshy fruit might need to be re-evaluated by more comprehensive genetic evidence. In fact, a recent study in tomato demonstrates the embryo lethality of the high-order *sleil* LOF mutant *sleil1 sleil2 sleil3 sleil4* but the viability of the upstream *slein2* LOF mutant, suggesting that SlEIL proteins must carry out, to some extent, SlEIN2-independent functions in tomato [68].

## Ethylene in fruit growth

Successful fertilization is followed by rapid cell division and expansion of the newly formed young fruits. While auxin and GA play predominant roles during cell division and expansion in developing fruits, other hormones also have the capacity to influence fruit growth, possibly through crosstalk with auxin and GA [12]. Among them, the role of ethylene in controlling fruit size in different species appears to be controversial.

Based on the larger rosettes, leaves, and petals of *Arabidopsis* mutants with impaired positive regulators of ethylene signaling, ethylene has long been recognized as a growth inhibitor [76]. The application of a high level of ACC to fertilized ovaries results in smaller tomatoes [67], while treatment with ethylene inhibitors leads to increased fruit size in apples [77], suggesting a negative role of ethylene in fruit growth control, similar to the discovery in *Arabidopsis*. In support of this notion, tomatoes that overproduce ethylene and those with enhanced ethylene signal also exhibit smaller fruit size [78, 79]. Surprisingly, when ethylene signaling was completely blocked, it was found that tomato *slein2*-KO mutant plants produced much smaller fruits. The tomato *SlEIL* high-order mutant *sleil1 sleil2 sleil3/SlEIL3 sleil4* also resulted in smaller fruits, indicating that ethylene also positively regulates fruit growth in tomatoes [68]. The suppressed fruit growth in *slein2* is mediated, at least partially, by the impaired auxin accumulation derived from developing seeds. This implies the existence of a regulatory axis involving ethylene-auxin crosstalk and fruit growth regulation [68]. To explain these seemingly inconsistent conclusions regarding ethylene’s function in controlling fruit growth, the authors proposed that an appropriate basal concentration of ethylene is optimal for fruit growth, while the absence or excess of ethylene production is inhibitory. Consistent with this proposal, it was found that cucumber mutants with either higher (*short fruit 1*, *sf1*) or reduced (*acs2*) ethylene production exhibit fewer cell divisions and shorter fruits than the WT [80]. Another study in melon suggests that ethylene acts as an inhibitor of cell division but as an activator of cell expansion during fruit elongation [81].

**Figure 4:**
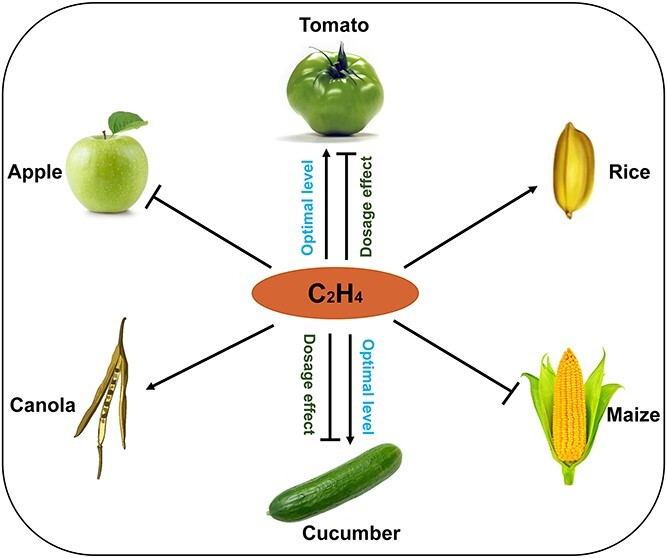
Schematic diagram demonstrating ethylene-mediated positive and/or negative regulation of fruit growth in six crop species. In particularl, the ethylene-mediated tomato and cucumber fruits’ growth requires an optimal ethylene level in a dosage-dependent manner. The regular arrow indicates positive regulation, and the ‘T’ represents negative regulation.

The inconsistency also extends to dry fruits. The *Arabidopsis* mutant with enhanced ethylene signaling, *eer5* (*ENHANCED ETHYLENE RESPONSE 5*), shows hypersensitivity to ethylene and shorter siliques [82]. In contrast, ACC-deaminase transgenic canola lines exhibit decreased ethylene content, resulting in smaller siliques and seeds [83]. Compared to other species with dry fruits, mounting evidence shows that ethylene promotes grain size in rice. The 1000-grain weights of *OsETR2* silencing lines are dramatically higher than those of the control, while *OsETR2* overexpressing plants show the opposite or unaltered effect [84]. In line with this, both the length and width of well-filled grains significantly decrease in *Osein2*/*mhz7* mutants, while overexpression of *OsEIN2/MHZ7* leads to increased grain length [85]. Similarly, the *Oseil1/mhz6* LOF mutant shows a remarkable decrease in grain length and width, while overexpression of *OsEIL1/MHZ6* increases grain size [86]. Further investigation has identified the rice OsEIL1-OsERF115-target gene regulatory module that controls grain size and weight by promoting longitudinal elongation and transverse division of spikelet hull cells, as well as enhancing grain-filling activity [87]. Interestingly, overexpression of *OsEIL2*, homologous to *OsEIL1*, results in smaller grain size [86]. Moreover, the characterization of a quantitative trait locus in maize, *qEL7* encoding ZmACO2, reveals a negative role of ethylene in controlling ear length [88]. Together, it seems that the role of ethylene in organ size control is much more complicated, implying that its functional characterization is highly dependent on the specificity of species as well as the tissue types (Fig. 4).

It has been shown that tomato and cucumber fruits with either higher or lower ethylene content display suppressed fruit growth [68, 80], suggesting that ethylene might function in a dosage-dependent manner. To maintain the optimal status, ethylene has evolved both autoinhibitory and autocatalytic mechanisms in climacteric fruits like tomato [9]. To operate the two contrasting ethylene generation systems at different developmental stages, some intrinsic molecular modules have been disclosed. For instance, several NAC and MADS-box transcription factors, which are specifically activated by tomato EIN3-like proteins at the ripening stage, are responsible for the ethylene burst required for the timely achievement of fully ripe fruits [68, 89]. Meanwhile, to avoid over-ripeness, other transcription factors, including SlAP2a, SlERF6, and SlMADS1, act as negative regulators of ethylene biosynthesis during tomato ripening [90–92]. The positive feedback regulation mediated by CsERF33-CsACS2 and CsERF110-CsACS11 modules has also been documented as a key molecular basis for initiating female flower development in cucumber [35, 36]. The transcription factor WIP1 plays a crucial role in regulating the level of ethylene during flower development in Cucurbits [93]. In order to produce female flowers, the expression of *WIP1* is suppressed by a high level of ethylene signaling in the carpel. Conversely, WIP1 hinders the development of male flowers by suppressing ethylene biosynthesis in the stamen [44]. Therefore, it is imperative to explore additional factors that contribute to the ethylene dosage effect in various plant species.

## Future perspectives

As a gaseous phytohormone, it remains poorly understood about how ethylene is confined to specific areas at an optimal level. The switching on or off of ethylene pathway genes in specific tissues dictates the formation of female or male flowers in cucurbits [26, 27]. Tomato fruit growth requires a relatively low level of ethylene, but a complete block of ethylene signaling leads to growth inhibition [68]. With the development of omics technology, particularly spatiotemporal transcriptome analysis, we can now differentiate transcripts not only of ethylene pathway genes but also their putative upstream regulators and downstream genes at the single-cell level. For instance, exploring the initiation site of floral sex determination in cucurbits, deciphering the switch between cell division and cell expansion at the early fruit growth stage, and uncovering the differences in ethylene perception in different cell types of fruits would become possible and informative in the near future.

The involvement of epigenetic regulation in fruit development and ripening is another important topic, as RNA modifications (e.g., m6A) and DNA modifications (e.g., 5mC) have been proven to be essential for fruit ripening in strawberry and tomato [94–96]. Moreover, it has been shown that transgenic expression of the human RNA demethylase FTO (fat mass and obesity-associated gene) in rice and potato results in a remarkable increase in yield [97]. During tomato fruit growth, a large number of fruit expansion-related genes are m6A modified and expressed more actively than the non-m6A-modified genes, suggesting a potential role of m6A modification in tomato fruit expansion [98]. Because ethylene plays a role in the entire life cycle of fruit development, the relationship between ethylene and epigenetic modifications remains to be explored. Additionally, the regulatory network of ethylene regulation in fruits responding to environmental factors such as light, temperature, and adverse conditions is far from understood, which would have high economic and agronomic value.
